# Complete Genome Sequence of Human Oral Saccharibacterium “*Candidatus* Nanosynbacter sp. HMT352” Strain KC1

**DOI:** 10.1128/mra.01205-21

**Published:** 2022-02-10

**Authors:** Karissa L. Cross, Dawn M. Klingeman, Mircea Podar

**Affiliations:** a Biosciences Division, Oak Ridge National Laboratory, Oak Ridge, Tennessee, USA; b Department of Biological Sciences, Vanderbilt University, Nashville, Tennessee, USA; Indiana University, Bloomington

## Abstract

*“Cand.* Nanosynbacter sp. HMT352” strain KC1 is an ectoparasitic saccharibacterium/TM7 that was co-isolated from a human saliva sample with its obligate bacterial host, *Schaalia odontolytica*. The genome of strain KC1 enables studies of the mechanisms and evolution of interspecies interactions and, for oral species, studies of their potential roles in health and disease.

## ANNOUNCEMENT

Saccharibacteria (originally referred to as candidate division TM7) are a group of ubiquitous bacteria which, based on their reduced genomes, are inferred to be physiologically dependent on acquiring metabolic building blocks from the environment or directly from other organisms (1–4). Several saccharibacteria have been cultured from the human oral microbiota as obligate ectoparasites on various Actinobacteria (5–9). An isolate from wastewater foam, “*Cand.* Mycosynbacter amalyticus,” attaches to and lyses a variety of free-living Actinobacteria (10). The mechanisms by which saccharibacteria recognize suitable hosts, acquire nutrients, and (in many cases) lead to host lysis are still unknown. By using a targeted reverse-genomics approach, we isolated and cultured a human oral saccharibacterium, “*Cand.* Nanosynbacter sp. HMT352” strain KC1 (here referred to as HMT352-KC1) in association with its host, the actinobacterium *Schaalia odontolytica* strain ORNL 0103 (6, 11).

HMT352-KC1 and its host were cultured in 100 mL brain-heart infusion medium (BHI, Difco) for 3 days at 37°C, under a hypoxic atmosphere (93% N_2_, 5% CO_2_, and 2% O_2_). All downstream molecular procedures used commercial reagents and followed manufacturers’ protocols. Genomic DNA was extracted using a Quick-DNA Fungal/Bacterial Midiprep Kit (Zymo Research). A Nextera XT DNA Library Preparation Kit (Illumina, Inc.) was used to generate a library with an approximately 600-bp median insert size, based on a Bioanalyzer 2100 (Agilent Technologies). The library was sequenced (2 × 250-nucleotide reads) on a MiSeq instrument (Illumina, Inc.), generating 11.1 million paired-end reads. All subsequent bioinformatic analyses were conducted using software default settings unless otherwise specified. The reads were imported into KBase (12) and trimmed, based on quality scores, using Trimmomatic v.0.36 (13). The trimmed reads were assembled using MEGAHIT v1.2.9 (14) with the meta-sensitive setting and a minimum contig size of 2 kb. The contigs were binned based on nucleotide composition and coverage depth using MetaBAT2 v1.7 (15), resulting in three bins. Two of the bins contained contigs with high G+C% (>56%), corresponding to the genome of the *Schaalia* host, which had been previously sequenced (11). The third bin was represented by a single 678,346-bp contig with a G+C of 43% and was classified as Saccharibacteria based on GTDB-Tk v1.7.0 (16). Genes encoding proteins and RNAs were predicted and annotated using Prokka 1.14.0 (17) and the contig was imported into Geneious Prime 2021.0.1 (18) for final curation. Inspection of the genes at the contig ends revealed an identical region. Mapping of the sequencing reads to that region in Geneious enabled identification of the nucleotide position unique to each end. Based on this, one of the repeating regions was removed and the ends were joined, resulting in a circular 677,938-bp chromosome with a G+C content of 42.9%.

Final genome annotation was conducted using the NCBI Prokaryotic Genome Annotation Pipeline (PGAP) v5.3 (19). The chromosome of HMT352-KC1 encodes 675 proteins, 42 tRNAs, one rRNA operon (23S, 1S6, and 5.8S genes), and 2 noncoding RNAs (ncRNAs). The genome was compared with those of other human Saccharibacteria/TM7 using FastANI0.1.2 (20). Based on an average pairwise nucleotide identity (ANI) of 83%, strain HMT352-KC1 is a closely related species to “*Cand.* Nanosynbacter lyticus” TM7X, the first isolated saccharibacterium (5).

### Data availability.

The *“Cand.* Nanosynbacter sp. HMT352” strain KC1 genome sequence has been deposited in GenBank under the accession number CP089520. The version described in this paper is the first version, CP089520.1. The Illumina reads have been deposited in SRA under the accession number SRR17194431.
